# Epilepsy in women of childbearing age: a focused review

**DOI:** 10.1186/s42466-025-00430-y

**Published:** 2025-10-06

**Authors:** Jan Heckelmann, Bettina Schmitz, Yvonne G. Weber, Catrin Mann

**Affiliations:** 1 Department of Epileptology and Neurology, RWTH Aachen University, Aachen, Germany; 2 Department of Neurology, Center for Epilepsy, Vivantes Humboldt Hospital, Berlin, Germany; 3 Epilepsy Center Frankfurt Rhine-Main, Department of Neurology, University Hospital Frankfurt (Main), Goethe University Frankfurt, Schleusenweg 2-16 (Haus 95), 60528 Frankfurt, Germany

**Keywords:** Seizures, Antiseizure medication, Pregnancy, Folic acid supplementation, Contraception

## Abstract

Epilepsy affects over 50 million individuals worldwide, a significant proportion of whom are women with epilepsy (WWE) of childbearing age. This population faces unique challenges related to hormonal fluctuations, e.g., during life stages such as breastfeeding or menopause. Antiseizure medications (ASMs) further complicate reproductive health by influencing menstrual function, contraception, pregnancy outcomes, bone health, and menopausal transition due to their teratogenic potential and hormonal interactive effects. Consequently, treatment strategies for WWE must consider these interactions and the risks associated with ASMs during pregnancy. This review aims to consolidate current data and guidelines for managing WWE throughout their reproductive years. These findings emphasize the importance of preconception counseling to optimize ASM regimens, ensuring both maternal well-being and fetal safety. Key recommendations from major international pregnancy registries are summarized to guide clinicians in selecting ASMs that minimize the risk of congenital malformations while maintaining effective seizure control. Additionally, this review explores the role of folic acid supplementation in preventing neural tube defects and outlines contraceptive options tailored for WWE. In conclusion, comprehensive education on the implications of epilepsy for reproductive health is crucial for WWE. By fostering informed decision-making through personalized counseling and careful medication management before, during, and after pregnancy, healthcare providers can significantly improve outcomes for both mothers and their children.

## Key points

**Table:** 

**Contraception**	
Birth control counseling should be provided before sexual activity.	
First recommended method of contraception is a copper or hormone coated IUD	
In case of ASM therapy avoid oral contraceptives in combination with strong CYP450-enzyme inducers.	
If oral contraception and ASM with interactive potential is used, double barrier is needed.	
In case of lamotrigine and oral hormonal contraception, changes of serum levels must be controlled.	
**Before pregnancy**	
Review epilepsy diagnosis.	
Clarify if ASM therapy is still necessary.	
Secure optimal ASM regimen.	
Monotherapy of lamotrigine or levetiracetam should be preferred.	
Rearrange total dosage of ASMs to 3 times intake per day.	
Determine blood levels of ASMs.	
Start folic acid with 0.4–0.8mg/d before pregnancy.	
**1st trimester of pregnancy**	
Control ASM serum level every 4 weeks.	
If the ASM serum drops more than 1/3 of previous level, elevate dosage of ASM and control serum level after 2 weeks.	
Adjust ASM dosage if seizures appear.	
In case of generalized seizures perform fetal ultrasound.	
Continue folic acid.	
**2nd trimester of pregnancy**	
Control ASM trough serum level every 4 weeks.	
Perform a specific ultrasound approx. in week 13 of pregnancy to early detect malformations.	
If the ASM serum drops more than 1/3 of previous level, elevate dosage of ASM and control trough serum level after 2 weeks.	
Adjust ASM dosage if seizures appear.	
In case of generalized seizures perform fetal ultrasound.	
**3rd trimester of pregnancy**	
See 2nd trimester.	
Advice patients and relative the aspects to have in mind for delivery and post-pregnancy phase.	
**Delivery**	
Vaginal spontaneous delivery can usually be attempted.	
A postnatal care by a midwife should be planned.	
Regular intake of ASMs should be secured.	
If seizures appear, benzodiazepines can be administered safely.	
**After pregnancy**	
Advice the patients and the relatives concerning secured handling of the baby (diapers change, bathing, carry around, avoid sleep depreviation).	
Advice patient and relatives that breastfeeding should be preferred.	
Doses of possibly elevated ASM should be reduced within 1–2 weeks after birth.	

## Introduction: Specific challenges in the treatment of women with epilepsy—from puberty, contraception, and pregnancy to menopause and beyond

More than 50 million people worldwide are affected by epilepsy, approximately 30–40% of whom are women of childbearing age [20]. Women with epilepsy (WWE) encounter unique challenges, including cyclical hormonal fluctuations, pregnancy, and breastfeeding periods. A bidirectional relationship exists between epilepsy, sex hormones, and the menstrual cycle: estrogen typically has proconvulsive effects, whereas progesterone and its metabolite allopregnanolone exhibit anticonvulsant properties. Variability in hormone levels during the menstrual cycle can exacerbate seizures, a phenomenon known as catamenial epilepsy [1, 26, 68]. On the other hand, some studies of cyclical seizure occurrence have indicated that seizure cycles are equally prevalent in men and women, with seizures displaying nonrandom cycles in general, including circadian, ultradian, lunar, multidien, and annual patterns and periodicities [24, 35, 39]. Thus, the prevalence of genuine, hormone-dependent catamenial epilepsy may have been overestimated previously. A trial of hormonal therapy in women with suspected catamenial epilepsy found no significant overall benefit; however, post hoc analyses identified a smaller subgroup that may have responded to treatment [28]. Neurosteroid withdrawal, mediated by the premenstrual decline in progesterone levels, increases seizure susceptibility in rodent models, diminishes benzodiazepine efficacy, and enhances the anticonvulsant potency of exogenous neurosteroids [54].

These results indicate that catamenial influences represent one component of monthly seizure variability in WWE rather than the dominant underlying mechanism.

Conversely, epileptic seizures may influence the female cycle by disrupting the hypothalamic-pituitary axis, leading to an increased incidence of menstrual irregularities and endocrine dysfunction in WWE [27]. Moreover, antiseizure medications (ASMs) can influence multiple aspects of reproductive health—including menstrual function, contraception, pregnancy, bone health, and menopause—due to their teratogenicity and enzyme-inducing potential [8, 67]. Optimal treatment strategies for WWE should include consideration of these interactions and the potential risks associated with ASMs prescribed during pregnancy. Accordingly, international recommendations for optimal therapy among women of childbearing potential and during pregnancy have been published [59, 60]. Since randomized trials are not feasible for ethical reasons, we must rely on the large pregnancy registries to assess the teratogenicity of ASM. These registries and the resulting recommendations on ASM use have contributed to a significant decline in congenital malformations among offspring of WWE over the past 25 years [3].

Owing to the possibility of unplanned pregnancies, and to avoid necessary medication changes in later phases of life, the safest possible ASM with respect to later pregnancies should be chosen for epilepsy treatment in young women at initial treatment. Individuals with epilepsy of reproductive age should receive comprehensive counseling that includes medication optimization, risk mitigation, and informed decision-making via person-centered approaches [5]. As part of the transition to adulthood, epilepsy-related education including sex-specific challenges must be provided to patients as early as possible, ideally during adolescence [10]. WWE continue to demonstrate inadequate knowledge regarding epilepsy and its interactions with pregnancy, parenthood, and hormonal balance [42]. Deficiencies in patient education among women of childbearing age may result in suboptimal therapeutic adherence during pregnancy, potentially compromising maternal and fetal outcomes. Pre-pregnancy counseling is essential to ensure that the woman enters pregnancy on the most appropriate ASM treatment, taking efficacy as well as fetal safety into account, and ensuring compliance and folic acid supplementation.

The aim of this short, focused review is therefore to present the current data and recommendations regarding the treatment and counseling of WWE of childbearing age. We place particular emphasis on management and appropriate medication during pregnancy by summarizing the findings of the major international pregnancy registries and the resulting recommendations of national guidelines. The management of WWE before, during and after pregnancy is described and the current evidence and possible risks associated with folic acid supplementation in WWE are discussed. Finally, the interactions of ASM with contraceptives and optimal contraceptive strategies are addressed.

## The teratogenic risk and safety profile of antiseizure medication

The optimal ASM regimen with the best safety profile should be initiated well in advance of a planned pregnancy [61]. The morbidity and mortality rates of WWE during pregnancy are slightly higher than those of the general population [41, 53]. This increase is partly due to reduced treatment adherence driven by patients’ concerns for the unborn child, and partly to diminished ASM efficacy caused by pregnancy-associated decreases in ASM blood levels. These declines can lead to a subsequent rise in seizure frequency and a greater risk of sudden unexpected death in epilepsy patients (SUDEP). Proactive ASM planning is also essential because children of WWE exposed to ASMs face an increased risk of malformations or cognitive impairments. The average risk of major congenital malformation (MCM), i.e. structural anomalies present at birth with significant medical, surgical, or cosmetic consequences, in children born to WWE on ASM treatment is 4.5% [3], whereas it is approximately 2.5% in the general population [37]. This significant difference cannot be attributed to epilepsy itself, since the risk of MCM in WWE not receiving ASM therapy is not significantly increased compared with the general population [7, 19]. Growing knowledge of ASM safety profiles with respect to the risk of MCM from pregnancy registries has led to changes in prescribing practices over recent decades, resulting in approximately 40% reduction in reported MCM rates [3, 60].

ASMs that should be strictly avoided include valproic acid (VPA), carbamazepine, phenobarbital, and phenytoin (Table 1). Dose-dependent MCM rates of up to 25% have been reported for these drugs, particularly for VPA at doses exceeding 1,450mg [3]. In addition, significant neurocognitive developmental delays and an increased incidence of autism have been described in children born to term who were exposed to VPA in utero [4]. Topiramate (TPM) is another ASM to avoid during pregnancy. TPM therapy appears to carry a significantly increased risk of cleft palate [25],cognitive developmental delays and autism spectrum disorders in exposed children [6]. Furthermore, in offspring of mothers with epilepsy, prenatal exposure to carbamazepine, oxcarbazepine, and TPM has been associated with an increased risk of being born small for gestational age (SGA); and CBZ has also been linked to a higher risk of microcephaly [11].

**Table 1:** Overview of pregnancy-related recommendations for common ASM (+ = recommended, – = not recommended, () = insufficient/inconsistent data)

ASM	Recommended during pregnancy	ASM level control recommended	CAVEAT
Carbamazepine	–	( +)	High risk for MCM
Eslicarbazepine	(o)	( +)	
Lacosamide	(o)	( −)	
Lamotrigine	+	+	
Levetiracetam	+	+	
Oxcarbazepine	+	+	
Phenobarbital	–	No change in regimen during pregnancy	High risk for MCM
Phenytoin	–	No change in regimen during pregnancy	High risk for MCM
Topiramate	–	( +)	High risk for cleft palate and developmental delays
Valproic acid	–	No change in regimen during pregnancy	High risk for MCM
Zonisamide	(o)	( +)	

In terms of the risk of MCMs, the recommended ASMs during pregnancy are levetiracetam (LEV) and lamotrigine (LTG) and, despite the possible risk of SGA, oxcarbazepine (OXC) is recommended [31]. In the most important registry and observational studies, none of these ASMs have been associated with a noticeable increase in MCM rates or, as far as has been investigated, with developmental delays [3, 25, 50]. A previously postulated dose restriction of LTG to 325mg daily during pregnancy is no longer necessary: recent registry data failed to demonstrate a significantly increased complication rate even above this dose [3].

The preferred approach during pregnancy is monotherapy with non-teratogenic ASMs, provided that therapeutic seizure control is maintained. When combination therapy is unavoidable, co-administration of LEV and LTG has not been associated with a significant increase in the risk of MCM [12, 64]. Recent evidence also suggests that much of the excess MCM risk observed with polytherapy can be attributed to high-dose valproic acid (VPA) use in earlier years [3].

Given that organogenesis and thus the phase of greatest susceptibility to relevant structural malformations occurs in the first 8–10 weeks of pregnancy, medication adjustments should be made prior to conception if pregnancy is anticipated [5]. As a significant proportion of pregnancies in WWE occur unintentionally, it is advisable that women of reproductive age be initiated on a pregnancy-compatible ASM as first-line therapy whenever possible.

As previously noted, ensuring adequate seizure control in WWE requires accounting for the decrease in ASM plasma concentrations during pregnancy, a phenomenon largely driven by increased hepatic metabolism and renal clearance [17, 49]. This is particularly relevant for LTG, LEV, TPM, zonisamide, or oxcarbazepine [30, 58], and applies even in the first trimester [49].

Therefore, a baseline ASM blood level should be determined before conception, and ASM blood levels should be monitored regularly in these patients. The optimal monitoring interval remains controversial: most experts advocate monthly checks, although some studies have not demonstrated clear benefits from such frequent testing [18, 48].

Considering that a reduction of more than 35% in ASM blood levels is associated with significantly worsened seizure control [55], it is advisable to establish a minimum target level of ASMs prior to pregnancy and to maintain this level by adjusting the dosage if serum concentrations fall below it. In cases of rapid or significant decline, monitoring should be performed more frequently than once per month [23]. Clear recommendations for individual dose adjustments are scarce in the current literature. Nevertheless, for women treated with LEV, a gradual dose escalation of 50% during the 1st trimester and up to 75% by the end of the 3rd trimester relative to baseline may be appropriate [17]. Recommendations for LTG remain neither clear nor uniform. Some authors advocate an initial dose increase of 20–25% above baseline, accompanied by monthly ASM plasma–level monitoring and repeat adjustments as levels fall [57]. Other studies, however, report substantial interindividual variability and caution against a standardized titration approach [2, 34].

Following delivery, clinicians must anticipate the rapid normalization of ASM metabolism and the ensuing risk of toxicity. Accordingly, the ASM dose should be reduced to pre-pregnancy levels within two weeks postpartum, guided by ASM level controls [30]. The dose reduction may be carried out in three steps every three days, each reducing approximately one third of the difference from the initial daily dose. However, evidence-based guidance on this approach is currently lacking.

Data on newer ASMs—such as lacosamide, zonisamide, and eslicarbazepine, which have become increasingly relevant in clinical practice in recent years—remain insufficient to support evidence-based recommendations in pregnancy.

In summary, careful planning and proactive management of ASM therapy are essential for WWE of childbearing age to minimize maternal and fetal complications. LEV or LTG should be prioritized as first-line treatments, with regular monitoring of serum levels and dose adjustments; combination therapy of LTG + LEV may be considered when monotherapy fails to achieve seizure control. Valproate should be strictly avoided whenever alternative therapies are available. Depending on reproductive intentions, either effective contraception or valproate dose reduction—potentially via transition to dual therapy with non-teratogenic ASMs—should be evaluated.

## Management and consultation of women with epilepsy before, during and after pregnancy

People with epilepsy (both women and men) have fewer children than people without this condition, primarily due to comorbidities and psychosocial barriers to finding a partner. Hormonal disorders that have been associated with epilepsy and that could affect fertility such as polycystic ovary syndrome appear to have only a minor impact on reproductive rates. In a study evaluating the time to conception among women planning pregnancy, no significant differences were found between those with and those without epilepsy [47].

Specialized counseling for WWE should begin early, no later than the onset of puberty. Counseling and guidance should be provided continuously by a designated contact person and, depending on the patient's age, by the pediatric neurology or specialized neurology departments. The transition of patient care should be ensured through a qualified transition process. Gynecological expertise should be integrated at every stage.

At each encounter, the following issues should be reviewed and discussed:

- The diagnosis should be reviewed, as the syndrome may evolve or change over time and alter counseling implications.
- Assessment of whether ongoing ASM therapy is required or if dose reduction or discontinuation is feasible
- Counseling regarding hormonal and/or contraceptive treatment and other additional medication, depending on the necessity to continue ASM.

In detail and depending on the age of the patients the following aspects should be considered:

During the preconception period—ideally six to twelve months before a planned pregnancy—it is essential to discuss the potential risks associated with ASM. This period should include a thorough review of the epilepsy diagnosis and an assessment of the ongoing need for ASM in general. The prescribed dosage itself should be critically reviewed with the total daily dose possibly divided into three equal administrations, depending on total dosages. Additionally, determining ASM serum levels is essential to ensure that therapeutic ranges are maintained. Prepregnancy seizure control, adherence to ASM therapy, and pregnancy planning are potential factors influencing both seizure control and delivery outcomes in WWE [45]. Furthermore, initiating folic acid supplementation is recommended to reduce the risk of fetal malformations [30].

In the first trimester of pregnancy, folic acid supplementation should be maintained, and a qualified ultrasound examination (i.e. DEGUM II) should be conducted around weeks 12–14 to check for any malformations. Although the rates of MCMs have declined over the past decade due to the established safety profiles of certain ASMs and improved clinical management, the average risk of MCMs in pregnancies among WWE remains elevated compared to the general population. Therefore, detailed prenatal ultrasound diagnostics may be offered to facilitate timely detection of MCM. Some anomalies may be amenable to intrauterine surgical intervention, while others require specialized perinatal management and prompt postnatal surgery. Importantly, following such diagnoses, some women may reconsider continuation of the pregnancy for medical reasons [32]. Beyond conventional obstetric guidelines, scientific evidence specifically addressing this issue in the epilepsy population is, to our knowledge, not available.

Monitoring ASM serum levels during this stage is also recommended, with the monitoring frequency tailored to the specific ASM. If levels are reduced significantly, an adjustment in ASM dosage may be needed. All pregnancies in women receiving ASM should be prospectively included in pregnancy registries by the treating neurologist (Box 1).

**Box 1:** Contact details for the EURAP Registry

International Homepage: EURAP—International Registry of Antiepileptic Drugs and Pregnancy
https://eurapinternational.org/
Eurap Germany:

https://www.dgfe.org/service/eurap#id4

As pregnancy progresses into the second trimester, an additional specialized high-resolution ultrasound examination should be performed at 19–22 weeks gestational age. Regular evaluation of ASM serum levels should be performed monthly.

During the third trimester, comprehensive counseling about childbirth becomes vital. In the absence of specific obstetric or neurological contraindications, delivery should be planned as a low-risk, uncomplicated vaginal birth. A planned cesarean delivery is not generally indicated. It is essential to ensure that regular intake of ASMs continues throughout labor, which can often last several hours. In the rare event that seizures occur during labor, benzodiazepines may be safely administered.

Postpartum, breastfeeding should be encouraged for a period of four to six months, on the basis of maternal preference. Although most ASMs do pass into breast milk, adverse effects such as fatigue in infants are uncommon [62]. Moreover, compared with formula, breast milk provides superior nutritional and immunological benefits. Following delivery, it is advisable to promptly taper any potentially increased ASM doses within approximately 1–2 weeks after delivery. The family should be counseled to implement appropriate modifications to newborn care. Parents with epilepsy should employ additional precautions: this includes avoiding prolonged carrying of babies; instead, breastfeeding and other caregiving activities should be performed while seated or reclined. Relatives should help prevent fatigue and sleep deprivation in WWE. Bathing infants should only be done in the presence of another adult for safety reasons.

In addition to puberty and pregnancy, perimenopause constitutes a further major hormonal transition in women with epilepsy that requires specialized counseling. Few data are available on epilepsy and the menopause. During this phase, women may experience previously unknown intolerances to ASMs [63, 69] or an exacerbation of seizure activity due to hormonal fluctuations [9, 59]. In such cases, close collaboration between neurologists and gynecologists is essential to evaluate treatment tolerability, adjust dosages as necessary, and ensure optimal management throughout this life transition.

## Folic acid supplementation in women with epilepsy

The positive effects of folic acid prophylaxis during pregnancy and the association between folic acid deficiency and MCM rates in the general population are well established [15]. Folic acid supplementation, which is recommended for all women of childbearing age, significantly reduces the risk of neural tube defects [14]. Folate deficiency is also associated with an increased risk of preeclampsia and preterm birth [33].

As outlined in the previous section, ASM can increase the risk of congenital anomalies, cognitive impairments, and other neuropsychiatric disorders in offspring of WWE. For certain ASM, these effects have been linked to maternal folate deficiency or antagonism. Small-scale and anecdotal studies have reported that the use of specific ASM—such as valproate, carbamazepine, lamotrigine, phenobarbital, and phenytoin—may lead to folate deficiency [40]. Additionally, evidence suggests that VPA may impair folic acid transport across the placenta and into the fetal brain [56]. Experimental animal and in vitro studies have demonstrated that VPA exposure can directly inhibit neural tube closure, an effect that appears reversible with high-dose folic acid supplementation [13, 46, 70].

With the aim of risk reduction, folic acid supplementation has been recommended for WWE for some time. The former guideline issued by the German Society of Neurology, which was valid until 2023, recommended a prophylactic dose of 5 mg/day [16]. However, the appropriateness of this high-dose recommendation has recently become the subject of considerable debate.

The rationale for high-dose folic acid prophylaxis in epilepsy is based primarily on a single study, which demonstrated that a daily dose of 4 mg reduced the recurrence risk of neural tube defects in subsequent pregnancies among women without epilepsy who had previously given birth to offspring with neural tube defects. Notably, this study did not include a comparison with lower doses [51]. The evidence regarding the efficacy and optimum dosage of folic acid supplementation in WWE undergoing ASM therapy remains inconsistent. To date, no robust clinical data exist to determine the optimal folic acid dosage for WWE receiving ASM, and a definitive reduction in MCM risk through folic acid prophylaxis in this population has not been demonstrated.

A cohort study published in 2022 from Scandinavia reported that among women with epilepsy treated with ASM, exposure to high-dose folic acid (≥ 1 mg/day; median 4.3 mg) during pregnancy was significantly associated with an increased risk of cancer in offspring up to 20 years of age, compared with those exposed to lower doses (< 1 mg/day) [65]. The same research group also reported an elevated risk of malignancies—particularly non-Hodgkin lymphomas—in women regardless of ASM use following intake of high-dose folic acid during pregnancy [66].

In light of emerging risk signals and the limited evidence supporting high-dose folic acid prophylaxis, current recommendations have been revised to a daily dosage of 0.4–0.8 mg [31]. Since neural tube closure defects typically occur between gestational days 21 and 28—often before pregnancy is recognized—folic acid supplementation should ideally begin before conception. Given the high incidence of unintended pregnancies among WWE, general folic acid supplementation at a dosage of 0.4 mg per day may be warranted for all WWE of childbearing potential.

In specific clinical scenarios, such as a positive family history of neural tube defects—particularly following the birth of a child affected by such a defect—or in cases involving the use of certain enzyme-inducing ASMs (e.g., carbamazepine) or VPA, folic acid dosages of 1–2.5 mg/day may be considered. Supplementation should be maintained at least through the end of the first trimester. Continued folic acid supplementation in pregnancy beyond the early period recommended to prevent neural tube defects may have beneficial effects on cognitive development of the child [43, 44], although data regarding the optimal duration of prophylaxis remain scarce.

## Contraception in women with epilepsy

Family planning counseling is a crucial component of care for WWE of childbearing potential, yet they still have lower rates of contraceptive use, in both high- and low income countries. Up to 30% of WWE of childbearing potential do not use highly effective birth control methods, and 7% may combine hormonal contraception with enzyme-inducing ASM, thereby risking contraceptive failure [21].

In a study conducted in the United States, two-thirds of all pregnancies among WWE were unplanned, often owing to ineffective contraceptive methods [29]. Several studies worldwide have demonstrate a striking and perstitent lack of knowledge with respect to contraception in this group [42], stressing the importance of early and repeated counseling.

Planning a pregnancy well in advance is particularly important for WWE to minimize treatment- and seizure-related risks. Therefore, contraception should be addressed at every consultation. Special attention is warranted for girls around puberty, patients with psychiatric comorbidities or intellectual disabilities, and those with syndromes associated with risk-taking and impulsive behavior—such as frontal lobe epilepsy and juvenile myoclonic epilepsy.

International preferences for contraceptive methods vary markedly. In Europe, there has been an ongoing trend toward a declining popularity of oral contraceptives (OC) in recent years, particularly among younger women. In Germany, for example, only 26% of women under 21 years of age used an OC in 2024, according to the Techniker Krankenkasse [38]. Positive attitudes among European healthcare providers and users regarding the safety and effectiveness of intrauterine devices (IUD) have contributed to their growing popularity compared with the US or Asia. In Europe, IUDs rank as the third most common contraceptive method after OCs and condoms.

IUDs are currently recommended as the preferred method of contraception for WWE, as they are safe, free of interactions with ASMs and unaffected by user error. Both copper and hormone-releasing IUDs are suitable. Although copper IUDs may increase bleeding and menstrual pain, they avoid the systemic hormonal side effects associated with hormone-releasing devices.

### Interactions between ASM and OC

Due to interactions, many ASMs (Table 2) compromise the contraceptive efficacy of hormonal OC. Older‐generation ASMs—such as carbamazepine, phenytoin, and barbiturates—induce CYP3A enzymes, thereby accelerating the hepatic metabolism of both estrogenic and progestogenic OC components, reducing serum hormone concentrations, and increasing the risk of contraceptive failure.

**Table 2:** Pharmacokinetic Interaction between ASM and OC. Adapted from [5]

	Serum levels of anti seizure medications reduced by oral contraceptives	Efficacy of oral contraceptives reduced by anti seizure medications
Brivaracetam > 100mg	No	YES
Cannabidiol	No	No
Carbamazepine	No	YES ↓↓↓
Cenobamate	No	YES ↓↓↓
Clobazam	No	No
Clonazepam	No	No
Eslicarabazepine	No	YES ↓↓↓
Ethosuximide	No	No
Felbamate	No	YES ↓↓↓
Gabapentin	No	No
Lacosamide	No	No
Lamotrigine	YES ↓↓↓	YES ↓ (progestin)
Levetiracetam	No	No
Oxcarbazepine	No	YES ↓↓↓
Perampanel > 8mg	No	YES
Phenobarbital	No	YES ↓↓↓
Phenytoin	No	YES ↓↓↓
Pregabalin	No	No
Primidone	No	YES ↓↓↓
Retigabine	No	No
Rufinamide	No	YES ↓↓ (estrogens)
Topiramate > 200mg	No	YES
Valproic acid	YES ↓	No
Zonisamide	No	No

Newer ASM, such as brivaracetam, cenobamate, topiramate and oxcarbazepine, exhibit low enzyme-inducing properties and may cause a selective reduction of progestin or estradiol concentrations in a dose-dependent manner. ASM that do not induce CYP3A such as lacosamide and LEV are not expected to substantially affect OC. However, large discrepancies in hormonal interaction reports are common with newer ASM. Notably, many OC–ASM combinations remain unstudied and the current assumption of interactions is often based on limited data or theoretical assumptions. Evidence regarding the interaction between OC and newer ASM, such as cannabidiol, is still scarce.

Importantly, interactions also apply to non-oral contraceptive hormones including injectable preparations, subdermal implants, and vaginal rings and may also diminish the effectiveness of emergency contraceptive pills („morning after pill“).

Conversely, combined OCs can affect the serum concentrations of some ASMs metabolized via glucuronidation, i.e. LTG, whose serum levels decrease by approximately 50% when taken with an OC, or conversely, increase when an OC is paused or discontinued. Glucuronidation is also crucial for the metabolism of VPA in combination with OC, and VPA concentrations can be reduced by 20–40% [22].

### Lamotrigine and oral contraceptives

Lamotrigine is the most frequently prescribed ASM for fertile WWE for two good reasons: LTG is recommended as first-line therapy both for focal and generalized epilepsies and it is not associated with teratogenic risk.

Bidirectional interactions between LTG and OC are complex and should always be discussed with patients before initiating therapy. LTG accelerates progestin clearance, reducing progestin concentrations by approximately 20%. Although this reduction may be clinically modest, it carries a risk of contraceptive failure—particularly with progestin-only OCs.

More importantly, combined OC decrease LTG concentrations. This effect is attributed to the estrogen component, which induces glucuronidation enzymes, thereby enhancing LTG metabolism and excretion. As a result, serum lamotrigine levels can decrease by up to 50%, potentially precipitating breakthrough seizures. Because combined OCs are administered in a 28-day cycle (21 days of active pills followed by 7 days of pill-free or placebo), lamotrigine concentrations decrease during the active pill phase and rebound during the pill-free interval. Earlier proposals that these interactions may be avoided by the use of an estrogen free pill have been challenged by more recent studies showing that certain progestin subtypes may also reduce LTG levels [52].

The effects of non-oral hormonal contraception on LTG pharmacokinetics are not well studied. However, non-oral hormonal contraceptives, such as estrogen patches and vaginal rings are likely to lead to substantial fluctuations in LTG concentrations upon initiation or withdrawal [36].

### Optimal contraception strategies

Contraception choices for WWE of childbearing potential should be individualized, taking into account potential interactions to minimize contraceptive failure and ensure optimal seizure control. Long-acting reversible contraceptives such as IUDs are both safe and effective. Both copper and levonorgestrel-releasing IUDs are recommended, since hormonal IUDs interact minimally with ASM. WWE receiving enzyme-inducing ASMs should be counseled that neither method of oral or non-oral hormonal contraception alone provides fully reliable contraception. A higher dose of oral contraceptives combinded with barrier methods, such as spermicide-covered condoms are recommended, but should not be considered reliable.

A shared decision-making approach is recommended to ensure that both individual and cultural preferences are considered in regard to acceptable methods of contraception (Box 2).

**Box 2:** Contraception in WWE (adapted from [31])

1. Birth control advice should be provided before the onset of sexual activity and reviewed regularly, particularly for those who take ASM with higher risk. In the case of late-onset epilepsy, information about contraception should be provided when the first ASM is prescribed
2. Contraceptive methods should be tailored to the needs and abilities of patients. Ensure close consultation with the attending gynecologist
3. When oral contraceptives and ASM are administered simultaneously, attention should be given to a possible reduction in contraceptive protection. Avoid oral contraceptives in combination with strong CYP450-enzyme inducers
4. For WWE, the recommended method of contraception is a copper or hormone coated IUD, which is the safest user-independent method of contraception
5. If ASM are used with weak, dose dependent or unclear interactions in combination with OC additional barrier methods (e.g. condoms) should be recommended
6. LTG and oral or non-oral hormonal contraception: Anticipate substantial serum level changes when starting, stopping or interrupting hormones. Consider alternative methods of contraception (IUP). Check serum levels of LTG and use OC with ultra long cyclus (without a pill-free interval after 21 days)

## Summary and conclusion

This focused review addresses the multifaceted challenges faced by WWE across key life stages, including puberty, contraception, pregnancy, menopause, and beyond. The review underscores the need for structured, expert driven counseling that evaluates the necessity of long-term ASM therapy and explores the potential for safe dose reduction or discontinuation. Continuously, ASMs and contraceptive methods should be checked for interactions. ASM regimens should be optimized well in advance of a planned pregnancy. This review highlights the teratogenic potential associated with certain ASMs that can increase the risk of major congenital malformations and neurodevelopmental disorders in offspring, while noting that several ASMs are considered safe during pregnancy. Standard-dose folic acid supplementation is recommended for women planning to become pregnant to reduce the risk of MCM. Additionally, counseling regarding breastfeeding practices is essential as most ASMs pass into breast milk but rarely cause adverse effects in infants.

In conclusion, effective management of WWE requires a holistic understanding of how this neurological condition intersects with female reproductive health across different life stages. Proactive planning must be prioritized—optimizing ASM therapy before conception is crucial for minimizing potential risks during pregnancy. Continuous therapeutic drug monitoring during pregnancy ensures both adequate seizure control and fetal safety.

Healthcare providers play a pivotal role in delivering personalized care through education and counselling aimed at addressing knowledge gaps among WWE regarding their condition's implications for reproductive health.

By adopting a comprehensive framework that encompasses informed decision-making around contraception, preconception planning, optimized ASM management during pregnancy, and postpartum care considerations, healthcare professionals can significantly improve outcomes for both mothers and their children while minimizing potential complications associated with epilepsy during critical periods such as pregnancy and beyond.

## Data Availability

Data sharing is not applicable to this article as no datasets were generated or analysed during the current study.

## Abbreviations

WWE: Women with epilepsy
ASM: Antiseizure-medication
MCM: Major congenital malformation
IUD: Intrauterine device
LTG: Lamotrigine
LEV: Levetiracetam
OC: Oral contraception
TPM: Topiramate
VPA: Valproate
